# Efficacy of three different budesonide treatments in Chinese preschool children with recurrent wheezing

**DOI:** 10.1038/s41598-022-21505-9

**Published:** 2022-10-11

**Authors:** Lu Li, Fan Zhang, Ping Sun, Jiangzhen Zheng, Tingting Chen, Tao Huang, Fang Wang, Ke Li

**Affiliations:** 1 Department of Pediatrics, First Affiliated Hospital of Nanchang University, Nanchang, 330006 Jiangxi China; 2 Department of General Surgery, First Affiliated Hospital of Nanchang University, Nanchang, Jiangxi China

**Keywords:** Clinical trials, Respiratory signs and symptoms

## Abstract

To explore and compare the clinical control of three atomized inhalation budesonide (BUD) regimens for Chinese preschool children with recurrent wheezing using Test for Respiratory and Asthma Control (TRACK) scores. A total of 474 preschool children with positive Modified Asthma Predictive Index (mAPI) were randomly assigned to a daily group (initially given inhaled BUD 1 mg once a day and assessed every 4 weeks; if symptom were well controlled for 12 weeks, the dose was reduced to 25–50% of the previous dose until afinal dose of 0.25 mg once a day, maintained until 52 weeks), an intermittent high-dose group (1 mg twice daily for 7 days starting early during a predefined respiratory tract illness) and an intermittent medium-dose group (0.5 mg twice daily as soon as they contacted allergens or experienced nasal congestion, a runny nose, cough or other suspicious respiratory symptoms and continuing until symptoms were reduced or risk factors were absent for 3 days) for 52 weeks of treatment. The TRACK questionnaire was administered every 4 weeks. When TRACK scores were ≥ 80, symptoms were considered to be controlled. The average TRACK scores of the three groups after treatment were significantly higher than those before treatment (P < 0.001). There were no significant differences in the average TRACK scores and control rate after treatment at every 4 weeks in the three groups (P > 0.05). Te number of systemic glucocorticoid courses, urgent care visits for wheezing, and wheezing episodes before and after treatment were significantly different within each of the three groups (P < 0.001), but not among the three groups (P > 0.05). In clinical treatment of children, one of the three treatment options can be selected according to the specific situation case of mAPI- positive recurrent wheezing children.

## Introduction

Nearly one-fifth of infants suffer from recurrent wheezing^1^. Correct identification of the risk of asthma in preschool children with recurrent wheezing and appropriate intervention can improve the prognosis of these children^2^. The Modified Asthma Predictive Index (mAPI) is considered a very useful predictor of asthma in children in this age group^3,4^.

Inhaled glucocorticoid is commonly used in clinics to treat and prevent wheezing attacks. Existing studies recommend long-term inhaled corticosteroid (ICS) treatment as the preferred initial treatment for preschool children at risk of asthma^5–8^. However, in the actual clinical treatment, many parents are worried about the side effects of long-term ICS use, asymptomatic wheezing attacks, and the lack of regular follow-up by researchers, resulting in low drug compliance^9^. Poor long-term inhalation compliance often results in poor clinical efficacy^8,10^.

Budesonide (BUD) is anICS designed for use in preschool children with wheezing. The intermittent high-dose atomization inhalation BUD regimen prevents the deterioration of the condition of children who have positive mAPI values and can significantly reduce drug exposure^11^. The latest guidelines in China have listed the regimen as an option for preschool children with recurrent wheezing, although the guidelines also note that attention should be paid to the potential adverse effects of repeated high-dose ICS^12^.

The members of this research team adopted an intermittent medium-dose BUD regimen for preschool children with recurrent wheezing in their previous study. This regimen also achieved good efficacy^13,14^.

The Test for Respiratory and Asthma Control in Kids (TRACK) is the first and only quantitative tool available domestically or abroad that is recommended for evaluating wheezing control in pre-schoolers from multiple dimensions, including damage and risk^15^.

In this study, we aimed to explore and compare the clinical control of three atomized inhalation BUD regimens for the treatment of preschool children with recurrent wheezing using TRACK, with the aim of establishing a basis for appropriate intervention regimens in these children.

## Materials and methods

### Patients

We enrolled children between the ages of 12 and 59 months who had positive mAPI values from January 1, 2015, to May 1 2019^16,17^ and the following criteria: (1) having ≥ 4 wheezing episodes in the past year; and (2) meeting at least 1 major criterion or at least 2 minor criteria. The major criteria include parental history of asthma, physician-diagnosed atopic dermatitis, and allergic sensitization to at least 1 aeroallergen. The minor criteria include wheezing unrelated to colds, peripheral blood eosinophils ≥ 4%, and allergic sensitization to milk, eggs, or peanuts. (Patient selection was strictly controlled by doctors who specialize in the diagnosis and treatment of children's respiratory diseases, especially asthma, and were familiar with the mAPI). The exclusion criteria were as follows: (1) having wheezing caused by organic lesions and mechanical factors; (2) having received more than six systemic glucocorticoids or having been hospitalized more than two times within 52 weeks; (3) having used other asthma control drugs; and (4) having guardians who did not know the child’s medical history or did not agree to participate in the study.

### Study design

This was a prospective randomized controlled study. The children were randomly allocated to three groups by an independent person using a computer-generated list of random numbers: 158 in the daily group, 158 in the intermittent high-dose group and 158 in the intermittent general-dose group. All children were followed for a 52-week treatment period. The patients were assessed every 4 weeks after treatment in the outpatient department or by phone. The specific treatment regimens were as follows:

The children in the daily group were initially given inhaled BUD 1 mg once a day. Their conditions were assessed every 4 weeks (the specific assessment items and control levels were graded according to the Global Initiative for Asthma, box 6–4; both good control and partial control were considered disease control^8^. If the symptom was well controlled for 12 weeks, the child’s dose was reduced to 25–50% of the previous dose until the final atomized inhaled dose was 0.25 mg once a day; this dose was maintained to 52 weeks. If the symptom was not controlled, the original dose was used continuously^7^.

The children in the intermittent high-dose group were given inhaled BUD 1 mg twice daily for 7 days if they had a cough, runny nose and other respiratory symptoms before the wheezing began^11^.

The children in the intermittent medium-dose group were given inhaled BUD 0.5 mg twice daily as soon as they contacted allergens or had nasal congestion, a runny nose, cough or any other suspicious respiratory symptoms. The drug was not stopped until the symptoms were reduced or risk factors were absent for 3 days^14^.

All three groups were treated with BUD suspension (Pulmicort Respules, Astra-Zeneca LP, Australia, 2 mL: 1 mg) with an air compression pump (PARI GmbH, Germany, PARI JuniorBOY SX).

The researchers explained to the participants’ families that wheezing children can be switched to other treatment options according to their condition and instructed them regarding administering the spray inhaler properly, keeping an asthma diary, and recording the symptoms and medication days and dosages.

During the treatment period, the guardians were directed to contact the researchers any time their child began wheezing. Acute phase treatment, including oxygen inhalation, airway clearing, atomized SABA inhalation, systemic glucocorticoids, intravenous antibiotics and symptom management, could be given under the direction of the researcher^3,8^. The original intervention plan was continued after the child’s condition was stabilized.

All methods in this article were performed in accordance with the relevant guidelines and regulations. This study has been registered in the Chinese Clinical Trial Registry,under registry code ChiCTR2000031893 (registration date: 2020/04/13). The study was approved by the Ethics Committee of the First Affiliated Hospital of Nanchang University (ethics number 2017-050), and written informed consent was obtained from the patients' guardians.

### TRACK questionnaire

The validity and reliability of the TRACK have been verified^18,19^. We used the original validated version of the TRACK. The TRACK questionnaire consists of five questions. The answers to the first three questions are based on the last four weeks, the fourth question is based on the last three months, and the fifth question is based on the last 12 months. The TRACK questions are as follows: (1) symptom frequency (wheezing, coughing and shortness of breath); (2) frequency of night awakenings due to symptoms; (3) frequency of interference of symptoms with the child’s activities; (4) frequency of use of rescue mediation in the last three months; and (5) systemic corticosteroid use over the past year. Each answer is scored from 0 to 20 points using a Likert scale, and total scores range from 0 to 100. The higher the score is, the better the respiratory and asthma control^15^.

### Outcome measures

The primary outcome measure was the TRACK scores. A professional researcher evaluated the TRACK questionnaire according to the records of the families of the children before treatment began and every 4 weeks thereafter. When TRACK scores are ≥ 80, respiratory problems are considered controllable^15^, and the percentage of people with TRACK scores ≥ 80 is the control rate.

### Secondary outcomes

The secondary outcome measures included the number of systemic corticosteroid courses (oral or intravenous), wheezing episodes, and urgent care visits for wheezing during the 52-week treatment period.

Treatment failure indicated that the wheezing was not under continuous control, a wheezing attack was serious enough to require tracheal intubation, or serious adverse reactions related to the treatment drugs occurred during the follow-up period.

### Statistical analysis

Descriptive statistics were used to summarize demographic and clinical characteristics. Categorical variables are presented using proportions and were analysed using the chi-square test. Continuous data are presented as the means ± standard deviations, and the Kolmogorov–Smirnov test was used to test the normality. Normally distributed data were analysed by variance analysis, while non-normally distributed data were analysed by the Kruskal–Wallis test or the Mann–Whitney U test. The indicators at different time points were tested using repeated measures analysis of variance, with two-sided α = 0.05 as the significance level. One-way analysis of variance was used to compare the three groups at each time point. All statistical analyses were performed with SPSS statistical software (version 22.0).

## Results

### Baseline characteristics

**Figure 1:**
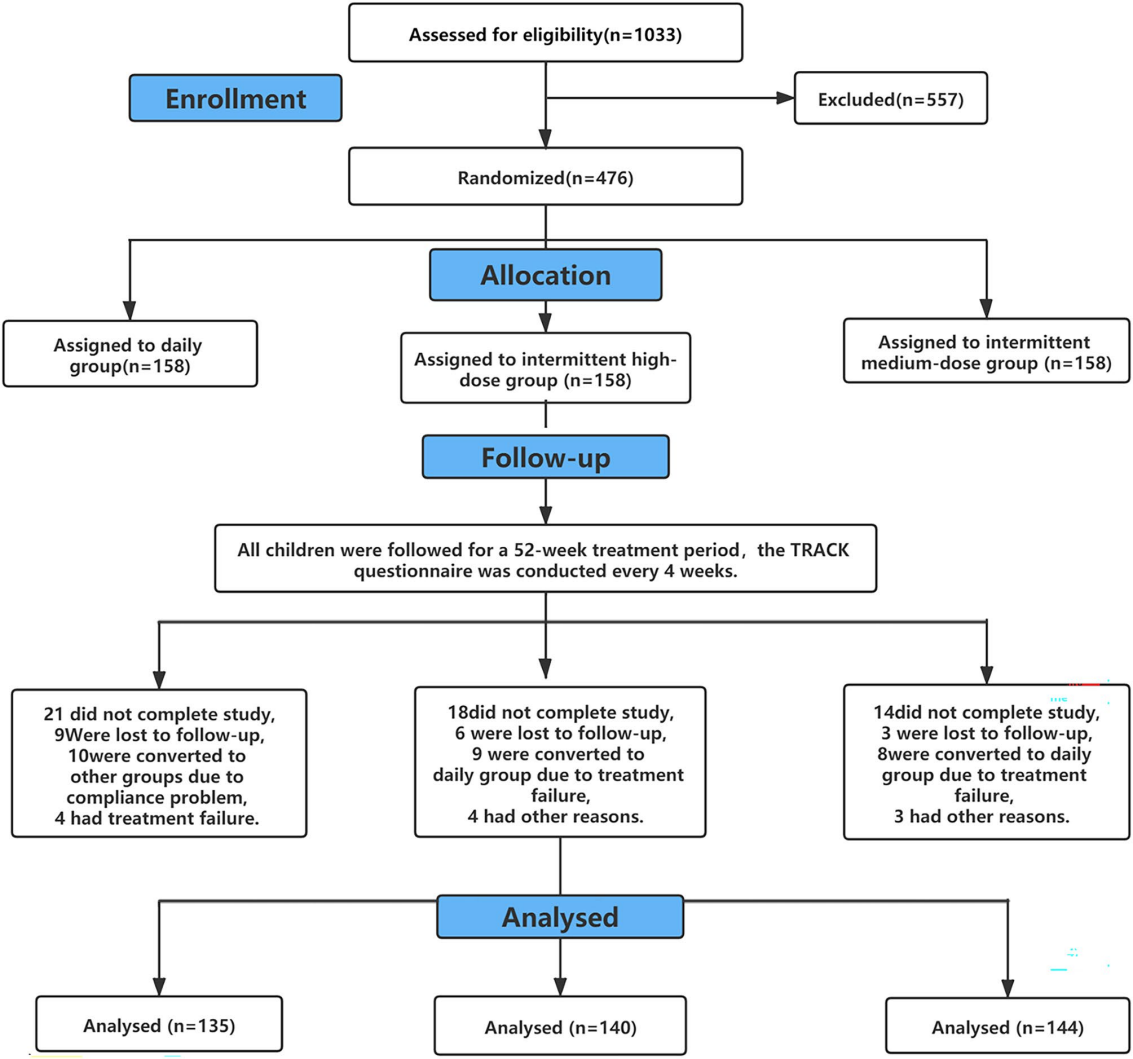
Study flowchart.

Of the 1033 children who were originally enrolled in the study, 557 were excluded from the study for various reasons: 281 had negative mAPI values; 39 had wheezing was caused by organic lesions and mechanical factors; 82 had received more than six glucocorticoids or had been hospitalized more than two times within 52 weeks; 54 had used other asthma control drugs; and 103 had guardians who did not know the child’s medical history or did not agree to participate in the study. A total of 474 children underwent randomization, and 419 completed the study (Fig. 1). The non-completion rate was 11.6%, with no significant differences among the three groups (P = 0.132). As shown in Table 1, there were no significant differences in the general information, breathing characteristics or specific background among the three groups (P > 0.05).

**Table 1:** Baseline characteristics of wheezing patients.

Group (N = 419)	Daily group (N = 137)	Intermittent high-dose group (N = 140)	Intermittent medium-dose group (N = 144)	P-value
Male sex—no. (%)	89 (65.0%)	96 (68.5%)	93 (64.6%)	0.738
Age (months)	29.26 ± 10.88	28.55 ± 10.38	29.53 ± 10.45	0.653^†^
No. of urgent or emergency visits over the past year	4.73 ± 1.17	4.57 ± 1.08	4.51 ± 1.00	0.131^***#***^
No. of wheezing episodes over the past year	5.33 ± 1.51	5.28 ± 1.42	5.22 ± 1.38	0.908^***#***^
Hospitalizations over the past year—no. (%)	60 (43.8%)	57 (40.7%)	58 (40.3%)	0.810
Patients who used systemic glucocorticoids over the past year—no. (%)	48 (35.0%)	45 (32.1%)	46 (32.6%)	0.829
No. of systemic glucocorticoid courses over the past year	0.44 ± 0.65	0.39 ± 0.61	0.40 ± 0.64	0.845^***#***^
Physician diagnosis of asthma—no. (%)	11(8.0%)	10 (7.1%)	9 (6.3%)	0.845
Any aeroallergen sensitivity	54 (39.4%)	52(37.1%)	57 (40.8%)	0.896
Food sensitivity	39 (28.5%)	47 (33.6%)	50 (34.7%)	0.494
Eosinophil percentage ≥ 4%—no. (%)	70 (51.1%)	69 (49.3%)	74 (51.4%)	0.930
Allergic rhinitis—no. (%)	18 (13.1%)	16 (11.4%)	20 (13.9%)	0.818
Eczema—no. (%)	92 (67.2%)	93 (66.4%)	98 (68.1%)	0.958
Parental asthma—no. (%)	13 (9.5%)	12 (8.6%)	11 (7.6%)	0.858

Categorical variables are presented using proportions and were analysed using the chi-square test.

Plus–minus values are means ± SD.

^†^One-way ANOVA.

^***#***^Kruskal–Wallis test.

### Outcome measures

The average TRACK scores in the daily group, the intermittent high-dose group and the intermittent medium-dose group at each time point from 4 to 52 weeks after treatment were significantly higher than those before treatment (P < 0.001).

The average TRACK score and the control rate for the three groups were significantly different between 4 and 12 weeks and between 8 and 12 weeks after treatment (P < 0.001), but the comparisons of adjacent time points between 12 and 52 weeks and between 4 and 8 weeks were not significantly different (P > 0.05).

There were no significant differences in the average TRACK scores at the same time point after treatment among the three groups (P > 0.05) (Table2).

**Table 2:** Comparison of the TRACK scores of the children in the three groups at different time points (mean ± SD).

Group (N = 313)	Daily group (N = 101)	Intermittent high-dose group (N = 105)	Intermittent medium-dose group (N = 107)	P-value^†^
0 weeks	42.63 ± 10.90	44.11 ± 11.62	44.10 ± 11.46	0.458
4 weeks	77.96 ± 3.76	78.25 ± 3.44	78.33 ± 4.14	0.685
8 weeks	77.99 ± 3.86	78.18 ± 4.16	78.58 ± 4.33	0.479
12 weeks	91.82 ± 10.27	91.36 ± 14.58	91.94 ± 13.22	0.921
16 weeks	91.02 ± 12.38	89.50 ± 16.94	91.32 ± 14.72	0.543
20 weeks	90.84 ± 13.29	89.21 ± 15.22	89.79 ± 15.60	0.650
24 weeks	89.45 ± 13.36	87.79 ± 16.66	89.44 ± 15.35	0.572
28 weeks	88.03 ± 15.40	88.29 ± 16.33	88.09 ± 16.95	0.991
32 weeks	87.52 ± 15.36	86.93 ± 17.51	89.17 ± 14.53	0.467
36 weeks	89.01 ± 13.56	88.43 ± 14.53	89.10 ± 16.51	0.920
40 weeks	89.85 ± 13.61	86.75 ± 18.00	90.35 ± 14.20	0.106
44 weeks	90.73 ± 13.03	89.43 ± 15.16	89.93 ± 15.55	0.758
48 weeks	90.84 ± 13.73	91.14 ± 11.93	90.73 ± 14.66	0.965
52 weeks	91.02 ± 14.74	91.82 ± 12.18	91.81 ± 12.75	0.848
P-value^††^	Group (F = 1.176) = 0.310			
	Time (F = 388.079) = 0.000			
	Group × time (F = 0.439) = 0.980			

There were no significant differences in the control rate at the same time point after treatment among the three groups (P > 0.05).

^†^Multivariate analysis of variance.

^††^Repeated measures ANOVA.

### Secondary outcomes

There were significant differences in the number of systemic glucocorticoid courses and urgent care visits for wheezing and wheezing episodes before and after treatment in the daily groups (P < 0.001).

There were significant differences in the number of systemic glucocorticoid courses, urgent care visits for wheezing and wheezing episodes before and after treatment in the intermittent high-dose group (P < 0.001).

There were significant differences in the number of systemic glucocorticoid courses and urgent care visits for wheezing and wheezing episodes before and after treatment in the intermittent medium-dose group (P < 0.001).

There were no significant differences in the number of systemic glucocorticoid courses or urgent care visits for wheezing and wheezing episodes during the 52-week treatment period among the three groups (P = 0.906, 0.143, and 0.621, respectively). There was no significant difference in the treatment failure rate among the three groups (P > 0.05) (Table 3).

**Table 3:** Secondary outcomes after treatment.

Group (N = 313)	Daily group (N = 101)	Intermittent high-dose group (N = 105)	Intermittent medium-dose group (N = 107)	P-value
No. of systemic glucocorticoid courses*	0.15 ± 0.36	0.14 ± 0.34	0.14 ± 0.35	0.906^†^
No. of urgent care visits for wheezing*	0.35 ± 0.48	0.41 ± 0.49	0.38 ± 0.49	0.621^†^
No. of wheezing episodes*	0.64 ± 0.53	0.77 ± 0.54	0.73 ± 0.63	0.143^†^
Patients who failed treatment—no./total no. (%)	4 (2.9)	9 (6.4)	8 (5.6)	0.378^††^

*Plus–minus values are means ± SD.

^†^Kruskal–Wallis test.

^††^Chi-square test.

### Safety

The average actual dose and duration of BUD use at 52 weeks for the patients in the daily group, intermittent high-dose group and the intermittent medium-dose groupwere 148.29 ± 18.55 mg and 299.19 ± 26.79 days, 57.75 ± 11.89 mg and 30.70 ± 6.64 days and 36.65 ± 6.74 mg and 35.30 ± 6.16 days, respectively.

**Figure 2:**
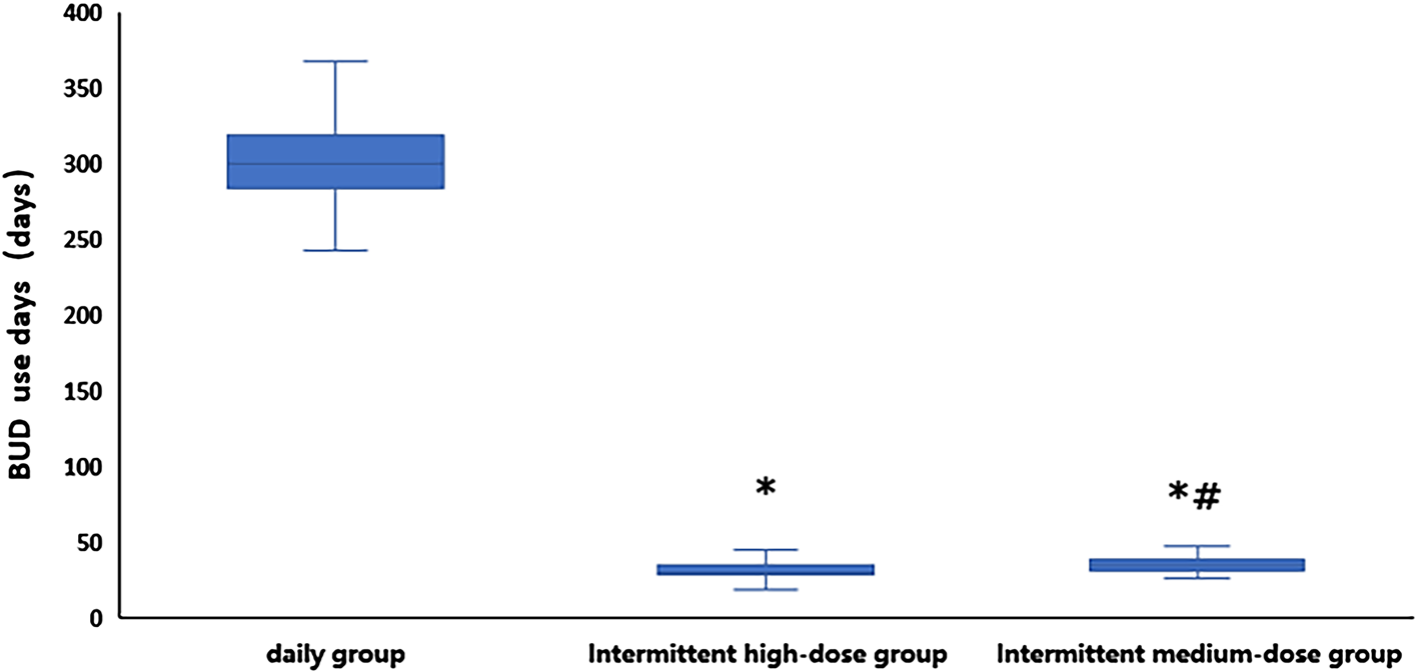
Comparison of the total days of actual BUD use in 52 weeks among the three group. *: Compared to the daily group, P < 0.001,^#^: compared to the intermittent high-dose group, P < 0.01.

The daily group had an obviously higher BUD dosage and more days of use than the intermittent high-dose group and the intermittent medium-dose group (P < 0.001). The BUD dosage of the intermittent high-dose group was higher than that of the intermittent medium-dose group, and the difference was statistically significant (P < 0.001), but duration of BUD use in the intermittent medium-dose group was higher than that in the intermittent high-dose group (P < 0.001) (Fig. 2). Neither death nor serious infections were reported in the three groups. In the daily group, 8 children developed mild thrush,and the BUD dosage of the intermittent high-dose group did not produce local adverse effects.

## Discussion

This study tried to explore and compare the clinical control of three atomized inhalation BUD regimens for the treatment of Chinese preschool children with recurrent wheezing using TRACK scores.

Zeiger et al. ^20^ showed that if the level of symptom control in children changed, the TRACK score differed by more than 10 points before and after treatment. A survey study found that the average TRACK score was highly reliable for assessing asthma control and changes in respiratory symptoms, 80 provided the best discrimination between children with controlled and uncontrolled respiratory symptoms^18^. The study results showed that with symptom control and over time, the average TRACK scores and control rates of the three regimens increased; they were significantly improved at 12 weeks after treatment and remained stable from 12 to 52 weeks after treatment,this further verifies that the TRACK can continuously reflect children's wheezing control status.

The study results also showed that all three BUD atomization regimens can reduce the need for systemic glucocorticoid treatment, the wheezing attack frequency, and the number of emergencies, with no statistically significant difference among the three groups. At the same time, there were no significant differences in the TRACK score, the TRACK control rate at all time points and clinical indicators after treatment with the three regimens. The results showed that the three treatment regimens can effectively control the symptoms of recurrent wheezing in preschool children with positive mAPI and have essentially the same results. This is consistent with our previous research results^14^.

However, the results showed that although the average TRACK scores at 4 and 8 weeks after treatment were improved compared with the scores before treatment, the control rate was still below 80%, which may be related to the risk domain determined the TRACK score, including the need for SABA in the past 3 months and the frequency of corticosteroid use in the past year^15^. This may lead to a low evaluation of the control situation, and if the child is treated with an upgraded treatment, the treatment may be excessive. Therefore, the use of the TRACK alone may lead to the underestimation of the level of symptom control, and it is necessary to perform a comprehensive evaluation of the condition that includes other clinical indicators^18,19^.

During the study, some of the children changed to another group due to treatment failure or compliance problems. These changes suggest the importance of individualized treatment. In clinical treatment, doctors should choose the appropriate treatment plan according to a comprehensive evaluation of the severity of the actual clinical wheezing of the child and the acceptance of the parents. At the same time, the three plans can be flexibly combined.

Moreover, mAPI positivity does not mean a diagnosis of asthma because a small number of children with positive mAPI values have not yet developed asthma; consequently, long-term ICS use may constitute a risk of overtreatment. In the actual clinical treatment, it is more appropriate to choose an intermittent regimen for children with mild wheezing, long intervals between atteacks or repeated wheezing after long-term atomization inhalation^20,21^, or to choose a daily atomized BUD inhalation regimen in the early stage and intermittent regimen in the later stage, which is also consistent with the GINA guidelines’s recommendation to employ gradual treatment according to the symptoms^8^. But this requires further follow-up study. This study excluded children who repeatedly used systemic corticosteroids and were hospitalized. Therefore, for children with frequent and severe wheezing attacks, a daily atomization inhalation BUD regimen is recommended^22,23^.

Intermittent high-dose atomized ICS may have potential side effects^2^. The latest research shows that dose and duration of ICS were significantly associated with adrenal suppression^24^. Based on this point, the modified TRACK scoring standard of China regards high-dose ICS use as the equivalent scoring standard for systemic corticosteroids^25^. If high-dose ICS is regarded as a criterion for systemic hormone equivalence, children who used inhaled BUD with high-dose atomization more than 4 times in the past 12 months will not be allowed to score. The number of children in the high-dose group whose TRACK scores are greater than or equal to 80 will be significantly reduced, and the feasibility of the scheme will be limited. In the actual clinical treatment, some children's symptoms disappear after 3 days of high-dose atomization, and some children's symptoms are still not controlled after 7 days of high-dose atomization; therefore, the 7-day therapy regimen is not flexible.

Another meta-analysis has shown that there is no difference in the amount and severity of deterioration between the two strategies when medium ICS is used in the intermittent design^2^. The children receiving the intermittent medium-dose regimen had an earlier administration time, the duration of administration was more flexible, and there were no potential side effects of repeated high-dose BUD inhalation. Additionally, family members were more accepting, making this regimen worthy of clinical promotion.

The limitations of this study are its exclusion of children with severe illness (the use of multiple hormones and multiple hospitalizations) and the use of a short-term treatment (52 weeks) without a longer effect and safety impact analysis.

In conclusion, the daily BUD atomized inhalation regimen, the intermittent high-dose atomized BUD inhalation regimen and the intermittent medium-dose atomized BUD inhalation regimen can significantly improve the clinical control of recurrent wheezing pre-schoolers with positive mAPI, and the control abilities of the three regimens are similar. In addition, the intermittent medium-dose nebulization inhalation BUD regimen has the earliest administration time, a flexible administration time and fewer doses, which can offer new options for pre-schoolers with recurrent wheezing and positive values on the mAPI. Clinical treatment can be based on the child’s specific situation when to choosing one of the treatment options, or a combination of multiple options.
